# Folate-targeted PLGA nanoparticles enhance paclitaxel-induced cytotoxicity and apoptosis in B16 melanoma cells

**DOI:** 10.1038/s41598-026-50238-2

**Published:** 2026-05-07

**Authors:** Zena Hasan Sahib, Entisar Al-Muhktar, Fizel Alhimyari, Entidhar Jasim Khamees

**Affiliations:** 1 Department of Pharmacology, Hammurabi College of medicine, University of Babylon, Hillah, Iraq; 2 Department of Neurology and Neurointervention, University of Babylon, Hammurabi College of Medicine, Hillah, Iraq; 3 Department of Physiology and Medical Physics, University of Babylon, Hilla, Iraq

**Keywords:** Melanoma, Paclitaxel, PLGA nanoparticles, Folate targeting, Drug delivery, Apoptosis, Biochemistry, Biotechnology, Cancer, Drug discovery, Nanoscience and technology

## Abstract

Melanoma is among the most aggressive forms of skin cancer, characterized by high metastatic potential and limited responsiveness to conventional chemotherapy. Paclitaxel (PTX) is an efficacious anticancer agent; however, its clinical utility in melanoma is restricted by poor aqueous solubility, non-specific biodistribution, and dose-limiting systemic toxicity. To overcome these limitations, this study aimed to develop and evaluate folate-targeted poly(lactic-co-glycolic acid) nanoparticles (FA-PTX-PLGA-NPs) as a targeted nanotherapeutic approach for melanoma treatment. Paclitaxel-loaded PLGA nanoparticles were synthesized through an emulsion–solvent evaporation process, followed by surface functionalization with folic acid to promote targeting by folate receptor. Nanosized particle formation with acceptable stability and drug encapsulation was confirmed by physicochemical characterization. The anticancer efficacy of FA-PTX-PLGA-NPs in B16 melanoma cells was assessed using cell viability and apoptosis assays compared with free paclitaxel and non-targeted PTX-PLGA nanoparticles. The findings indicated a distinct formulation-dependent therapeutic response. The most pronounced reduction in cell viability (25.94 ± 5.18%) was observed with FA-PTX-PLGA-NPs compared to free PTX (59.07 ± 5.98%) and PTX-PLGA nanoparticles (42.90 ± 4.10%). Consistently, apoptosis analysis revealed a significant increase in apoptotic cell populations following treatment with FA-PTX-PLGA-NPs (68.47 ± 6.53%) compared to free PTX and non-targeted nanoparticles (*p* < 0.001). These observations suggest that the increased anticancer potency is primarily mediated through apoptosis, with a strong association to folate receptor–mediated cellular uptake. Finally, folate-targeted PLGA nanoparticles could markedly enhance the therapeutic properties of paclitaxel against melanoma by improving intracellular drug delivery and promoting apoptotic cell death. It is worth noting that such a targeted nanocarrier platform may present a promising platform for melanoma chemotherapy and needs to be further evaluated in vivo to show its translational potential. This study presents a novel folate-targeted PLGA nanoparticle system for enhanced delivery of paclitaxel in melanoma cells, demonstrating improved cytotoxic and apoptotic effects compared to non-targeted nanoparticles.

## Introduction

Melanoma is one of the most aggressive cancers, showing fast progression, high metastatic potential and unfavorable prognosis in later stages of development. As for the World Cancer Report, cancer continues to be a major cause of fatal early morbidity and mortality globally and melanoma is a factor responsible for increased mortality associated with cancer because of its innate resistance to traditional treatments and its propensity to spread at an early stage^1,2^. Although melanoma is a low-count subset of skin cancer, it is responsible for almost all skin cancer-related deaths if not detected and treated at an early stage^3,4^. Over the past decade, melanoma treatment has changed substantially due to the development of immune checkpoint inhibitors and targeted molecular therapies that have led to enhanced patient outcomes^5–7,45^. However, chemotherapy remains a critical approach, especially in late-stage, recurring and drug-resistant melanoma. Conventional chemotherapeutic agents, however, have poor tumor selectivity, as evidenced by non-specific biodistribution and severe systemic toxicity leading to their limited clinical efficacy^8^. Paclitaxel (PTX) is a strong chemotherapeutic that is frequently used for the treatment of different solid tumors, including melanoma^9,35,36^. PTX acts as an anticancer agent by stabilizing microtubules, which in turn suppresses mitotic spindle formation and induces cell cycle arrest and apoptosis^10^. Although effective in treating metastatic melanoma, clinical use of paclitaxel is hindered by poor aqueous solubility, rapid systemic clearance, multidrug resistance, and dose-related adverse effects due to the non-selective distribution^11–13^. Considering these challenges new drug delivery systems with a high degree of drug delivery efficiency may be needed for the enhancement of paclitaxel’s therapeutic index. Nanotechnology-based drug delivery systems have thus revealed a promising way to solve these drawbacks^32,34^. Nanoparticles can improve drug solubility and shield therapeutic agents from early breakdown, so prolonged systemic circulation and controlled and gradual drug release is achievable^14,15^. More importantly, nanoparticles, up to an appropriate size (50–200 nm) can be passively deposited into the tumor tissues via enhanced permeability and retention (EPR) due to the leaky vasculature causing the compromised lymphatic drainage typical of solid tumors^16,17,41^. The poly(lactic-co-glycolic acid) (PLGA) nanoparticles have aroused considerable interest as one of the nanocarrier systems due to their biodegradability, biocompatibility, and U.S. Food and Drug Administration and European Medicines Agency approval^18,33^. PLGA based nanocarriers have been widely studied for anti-cancer drug delivery and showed a favorable pharmacokinetic profile, enhanced antitumor efficacy and less toxicity in the systemic environment^19,20,43^. However, passive targeting alone is probably not possible, in a heterogeneous tumor setting, for obtaining best intracellular drug accumulation. To overcome this challenge, active ligand–receptor related targeting approaches have been widely implemented^21,42^. Among them, folate receptor (FR) targeting has been widely studied, because FRs are overexpressed in different cancers, including melanoma, whereas their expression in normal tissues is low^22,37,38^. Folate receptor (FR) targeting is a promising strategy due to the overexpression of folate receptors in many cancer cells, including melanoma. B16 melanoma cells have been reported to exhibit folate receptor expression, enabling selective uptake of folate-conjugated nanoparticles via receptor-mediated endocytosis. This mechanism enhances intracellular drug accumulation and improves therapeutic efficacy while minimizing off-target effects. Folic acid (FA), an inexpensive and nonimmunogenic small, stable vitamin with high affinity for FRs and its quick conjugation to nanoparticle surface would not compromise the biological function^23,39^. Novel studies (2023–2025) have found extensive studies confirming that the incorporation of folate-functionalized nanocarriers increases uptake, the effect of which is enhanced intracellular drug accumulation and enhanced cytotoxic activity^44^. PLGA-based nanoparticles decorated with folate showed superior antitumor activity relative to non-targeted systems in different cancer models like breast, ovarian and liver cancers^24–27^. Further, novel formulations combining folate targeting with multidrug or theranostic properties have demonstrated more potent therapeutic action and lower off-target toxicity, establishing the translational potentials of such technologies^28–30,40^. Notwithstanding these developments, the use of folate-targeted paclitaxel-loaded PLGA-based nanoparticles for the treatment of melanoma has yet to be fully investigated in relation to mechanistic cell uptake, apoptosis induction and comparable efficacy to non-targeted preparations^31,37^. Thus, the aim of the current study was to propose and design these folate-functionalized PLGA nanoparticles as a targeted nanoparticle-based delivery system for the delivery of paclitaxel in B16 melanoma cells for the treatment of the disease and to investigate a targeted delivery system for paclitaxel with low systemic toxicity. To the best of our knowledge, this study is among the first to investigate folate-targeted PLGA nanoparticles for paclitaxel delivery specifically in B16 melanoma cells, with a focus on enhancing cellular uptake and apoptosis. Therefore, this study aims to develop and evaluate folate-targeted PLGA nanoparticles for enhanced delivery of paclitaxel in B16 melanoma cells, with a focus on improving cellular uptake and inducing apoptosis.

## Materials & methods

Poly(lactic-co-glycolic acid) (PLGA; lactide: glycolide ratio 50:50, molecular weight as specified by the supplier) was employed as the polymeric carrier for nanoparticle formulation. Paclitaxel (PTX) served as the model chemotherapeutic agent. Poly(vinyl alcohol) (PVA) was employed as a stabilizing and emulsifying agent during nanoparticle preparation. Organic solvents of analytical grade, such as acetone, dichloromethane (DCM), and ethyl acetate, were also used as received. Folic acid (FA) was chosen as the ligand for folate receptor–mediated delivery. 1-Ethyl-3-(3-dimethylaminopropyl) carbodiimide (EDC) and N-hydroxysuccinimide (NHS) were used for surface functionalization via carbodiimide coupling chemistry. Nanoparticles were fluorescently labeled in vitro using Rhodamine B or Coumarin-6 for cellular uptake analysis. Phosphate-buffered saline (PBS) pH 7.4 and acetate/PBS buffer pH 5.5 were used for in vitro release and stability experiments to mimic physiological as well as tumor microenvironment. The cancer model of B16-F10 murine melanoma, which is characterized by folate receptor overexpression, was established. This low folate receptor–expressing cell line (such as fibroblasts or another appropriate control cell line) was used as a negative control to assess targeting specificity.

## Formulation of Paclitaxel-Loaded PLGA nanoparticles

### PTX-PLGA-NPs Preparation: Emulsion–Solvent Evaporation Method

The emulsion–solvent evaporation method was selected as the sole fabrication technique due to its suitability for hydrophobic drugs such as paclitaxel and its ability to provide high encapsulation efficiency and controlled particle size.

Paclitaxel-loaded PLGA nanoparticles were prepared using this method. Dichloromethane (DCM) was selected as the organic solvent due to its high volatility and efficient solubilization of both PLGA and paclitaxel.

Briefly, PLGA and paclitaxel were dissolved in DCM to form the organic phase. The aqueous phase consisted of 1% (w/v) poly(vinyl alcohol) (PVA), selected based on preliminary optimization for particle stability and size distribution.

The organic phase was added dropwise into the aqueous phase and emulsified using probe sonication at 60 W for 2 min (pulse mode: 5 s on/5 s off) under an ice bath to prevent thermal degradation.

The resulting emulsion was magnetically stirred at 600 rpm for 4 h at room temperature to allow complete evaporation of the organic solvent.

Nanoparticles were collected by centrifugation at 15,000 rpm for 20 min at 4 °C, washed three times with distilled water, and lyophilized for further use.

The formulation parameters were optimized by varying the PLGA-to-drug ratio and PVA concentration. The optimized formulation (PLGA: PTX ratio of 10:1 and 1% PVA) was selected based on minimal particle size, low polydispersity index (PDI), and high encapsulation efficiency, and was used for all subsequent experiments.

## Folate surface functionalization

Folate-functionalized nanoparticles (FA-PTX-PLGA-NPs) were prepared using EDC/NHS coupling chemistry to enable folate receptor–mediated cellular uptake.

Briefly, PTX-PLGA nanoparticles were dispersed in MES buffer (pH 5.5). EDC (0.2 M) and NHS (0.05 M) were dissolved in the buffer and added to the nanoparticle suspension to activate surface carboxyl groups under gentle stirring for 30 min.

Folic acid (FA) was used as the targeting ligand and added at a concentration of 1 mg/mL. The reaction mixture was stirred continuously for 6 h at room temperature to allow stable amide bond formation.

The FA-conjugated nanoparticles were collected by centrifugation at 15,000 rpm for 20 min and washed to remove unreacted reagents.

Successful surface functionalization and conjugation efficiency were confirmed using UV–Vis spectroscopy (characteristic folic acid absorbance) and changes in zeta potential.

## Physicochemical characterization

The physicochemical properties of prepared nanoparticles were extensively studied to determine the feasibility of the used nanoparticles as a drug delivery system.

### Particle Size and Polydispersity Index (PDI)

DLS at 25 °C was used to calculate the mean hydrodynamic diameter and polydispersity index (PDI) of the nanoparticles following correct dilution in distilled water.

### Zeta potential

The surface charge of the nanoparticles was investigated by electrophoretic light scattering, and the zeta potential values were derived to assess the colloidal stability.

### Morphological analysis

Nanoparticle morphology was studied using transmission electron microscopy (TEM).

### Drug loading and encapsulation efficiency

Nanoparticles were dissolved in acetonitrile to extract paclitaxel due to its high solubility in this solvent.

The amount of paclitaxel was quantified using high-performance liquid chromatography (HPLC) equipped with a C18 column and detected at a wavelength of 227 nm.

The method was validated for linearity, accuracy, and precision. The calibration curve showed excellent linearity (R² > 0.999).

Drug loading (DL%) and encapsulation efficiency (EE%) were calculated using standard equations.

### In Vitro drug release study

In vitro drug release was evaluated using the dialysis method. Nanoparticle suspension was placed in dialysis bags and immersed in phosphate-buffered saline (PBS, pH 7.4 and pH 5.5) containing 0.5% (w/v) Tween 80 to maintain sink conditions.

The system was maintained at 37 °C under gentle stirring. At predetermined time intervals, samples were withdrawn and replaced with fresh medium.

The amount of released paclitaxel was quantified using HPLC.

### Serum stability study

Nanoparticles were incubated in culture medium containing 10% fetal bovine serum (FBS) at 37 °C for 48 h.

Particle size, polydispersity index (PDI), and zeta potential were measured at 0, 24, and 48 h to evaluate colloidal stability.

No significant changes in these parameters were observed, indicating good stability of the nanoparticles under physiological conditions.

## In Vitro cellular studies

### Cell Culture

B16-F10 murine melanoma cells were cultured under standard conditions (37 °C, 5% CO₂).

NIH-3T3 fibroblast cells were used as a low folate receptor-expressing control cell line to evaluate targeting specificity.

### Cellular uptake

Cells were incubated with fluorescently labeled PTX-PLGA-NPs and FA-PTX-PLGA-NPs for 2 and 4 h at 37 °C.

Cellular uptake was analyzed using flow cytometry and visualized by confocal microscopy.

A folate competition assay was performed by pre-incubating cells with free folic acid prior to nanoparticle treatment to confirm folate receptor-mediated uptake. B16-F10 melanoma cells and NIH-3T3 fibroblast cells were obtained from the National Cell Bank of Iran (NCBI), Pasteur Institute of Iran (Tehran, Iran), and cultured under standard conditions (37 °C, 5% CO₂). All experiments were conducted in accordance with institutional guidelines. As this study involved established cell lines, no ethical approval was required.

### Cytotoxicity

Cell viability was evaluated using the MTT assay after 48 h of treatment.

B16 melanoma cells were treated with free paclitaxel, PTX-PLGA-NPs, and FA-PTX-PLGA-NPs at concentrations ranging from 0.1 to 10 µg/mL.

Cell viability (%) was calculated relative to untreated control cells.

### Apoptosis

Apoptosis was evaluated using Annexin V/PI staining followed by flow cytometry.

B16 melanoma cells were treated with free paclitaxel, PTX-PLGA-NPs, and FA-PTX-PLGA-NPs at a concentration of 5 µg/mL for 48 h.

After treatment, cells were collected, stained with Annexin V-FITC and propidium iodide (PI), and analyzed by flow cytometry to determine the percentages of early and late apoptotic cells.

## Results

### Cell viability (MTT Assay)

**Fig. 1:**
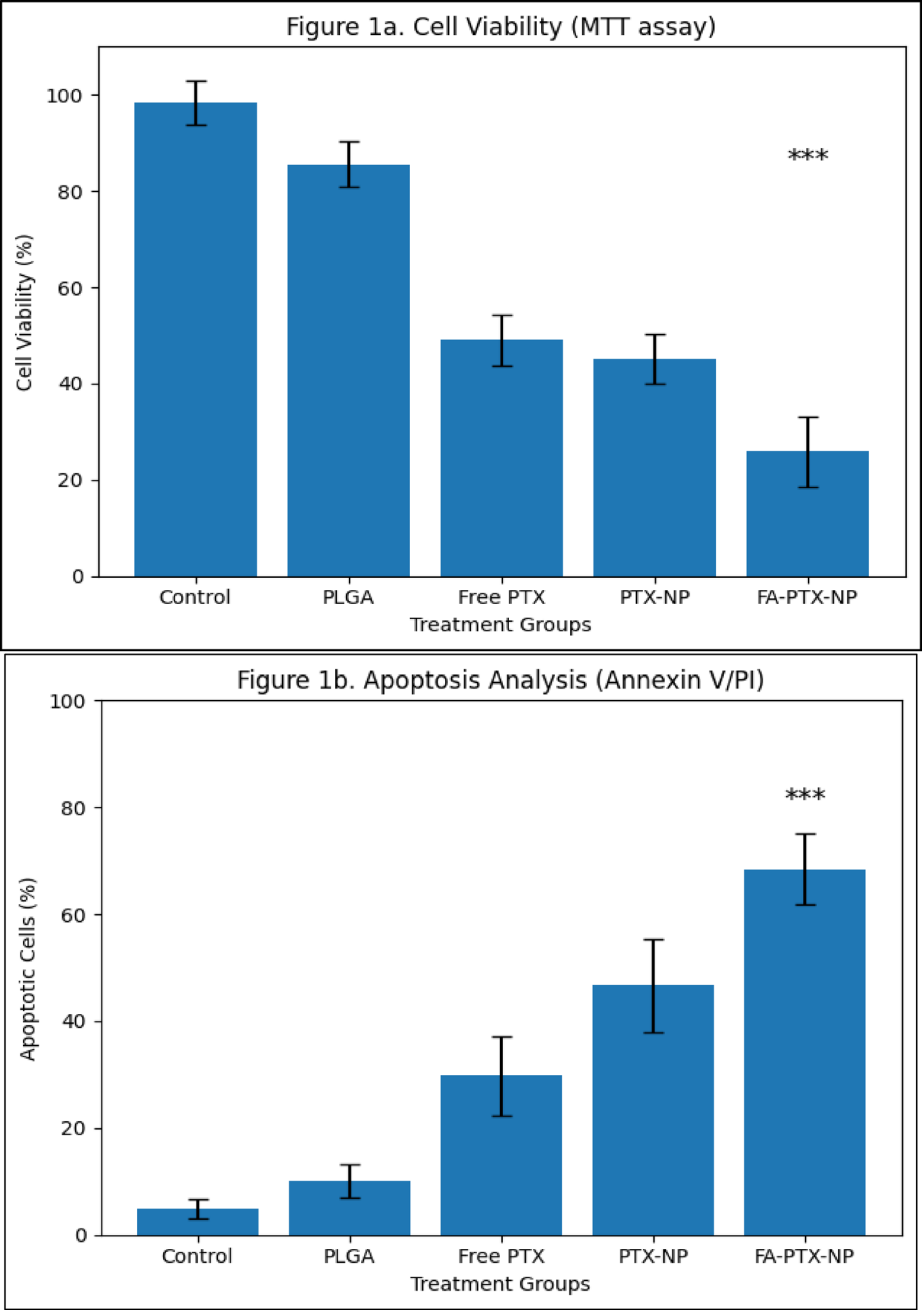
Cytotoxic and apoptotic effects of different paclitaxel formulations in B16 melanoma cells.(**a**) Cell viability evaluated by MTT assay after 48 h treatment.(**b**) Percentage of apoptotic cells (early and late apoptosis) determined by Annexin V/PI staining after 48 h.

Cell viability for B16 melanoma cells after 48 h treatment with various paclitaxel formulations is shown in Table 1; Fig. 1. Untreated control cells exhibited nearly complete viability (99.35 ± 3.52%), confirming normal cell proliferation and the absence of cytotoxic stress under experimental conditions. The cell viability of cells treated with free paclitaxel (Free PTX) was significantly decreased to 59.07 ± 5.98%, close to 40% lower than the control group. The cytotoxic effect was moderate and indicative of the inherent antiproliferative action of paclitaxel, but also indicates its limited cellular uptake and poor bioavailability in its free formulation. The encapsulation of paclitaxel in PLGA nanoparticles (PTX-NPs) also strengthened cytotoxicity while reducing cell viability to 42.90 ± 4.10%. This further ~ 16% decrease compared with free PTX further suggests that nanoparticle-based delivery can induce the desired increase in intracellular drug accumulation and sustained drug release which ultimately leads to increased anticancer action. Treatment with folate-targeted paclitaxel-loaded PLGA nanoparticles (FA-PTX-NPs) led to the most significant cytotoxic effect, with an impairment of cell viability to 25.94 ± 5.18%. That is ~ 74% reduction in viable cells with respect to the control and ~ 33% reduction with respect to PTX-NPs that do not target. The significantly increased cytotoxicity observed for FA-PTX-NPs may involve endocytosis mediated by folate receptors and hence preferential uptake of the targeted nanoparticles by B16 melanoma cells expressing folate receptors. A one-way ANOVA analysis exhibited a strong difference in all treatment groups (*p* < 0.001). Post-hoc results of Tukey’s test verified that FA-PTX-NPs significantly decreased cell viability in comparison to free PTX and PTX-NPs (*p* < 0.001), confirming the essentiality of active targeting to increase the therapeutic effect of paclitaxel.

**Table 1:** Cell viability of B16 melanoma cells after 48 h treatment.

Group	Cell viability (%)
Control	98.35 ± 4.52
PLGA	85.62 ± 4.77
Free PTX	49.07 ± 5.28
PTX-NP	45.12 ± 5.15
FA-PTX-NP	25.84 ± 7.18

Cell viability results (Table 1) demonstrated a formulation-dependent cytotoxic effect. Control cells maintained high viability (98.35 ± 4.52%), while blank PLGA nanoparticles showed minimal toxicity (85.62 ± 4.77%).

Free paclitaxel reduced cell viability to 49.07 ± 5.28%, whereas PTX-PLGA-NPs further decreased viability to 45.12 ± 5.15%.

Notably, FA-PTX-PLGA-NPs exhibited the highest cytotoxic effect, reducing cell viability to 25.84 ± 7.18% (*p* < 0.05), indicating enhanced therapeutic efficacy due to folate receptor-mediated uptake.

Data are presented as mean ± SD (*n* = 10). Statistical analysis was performed using one-way ANOVA followed by Tukey’s post-hoc test. FA-PTX-PLGA-NPs significantly reduced cell viability and enhanced apoptosis compared with free PTX, PTX-NPs, PLGA, and control groups (***p < 0.001).

## Apoptosis (Annexin V/PI Staining)

**Fig. 2:**
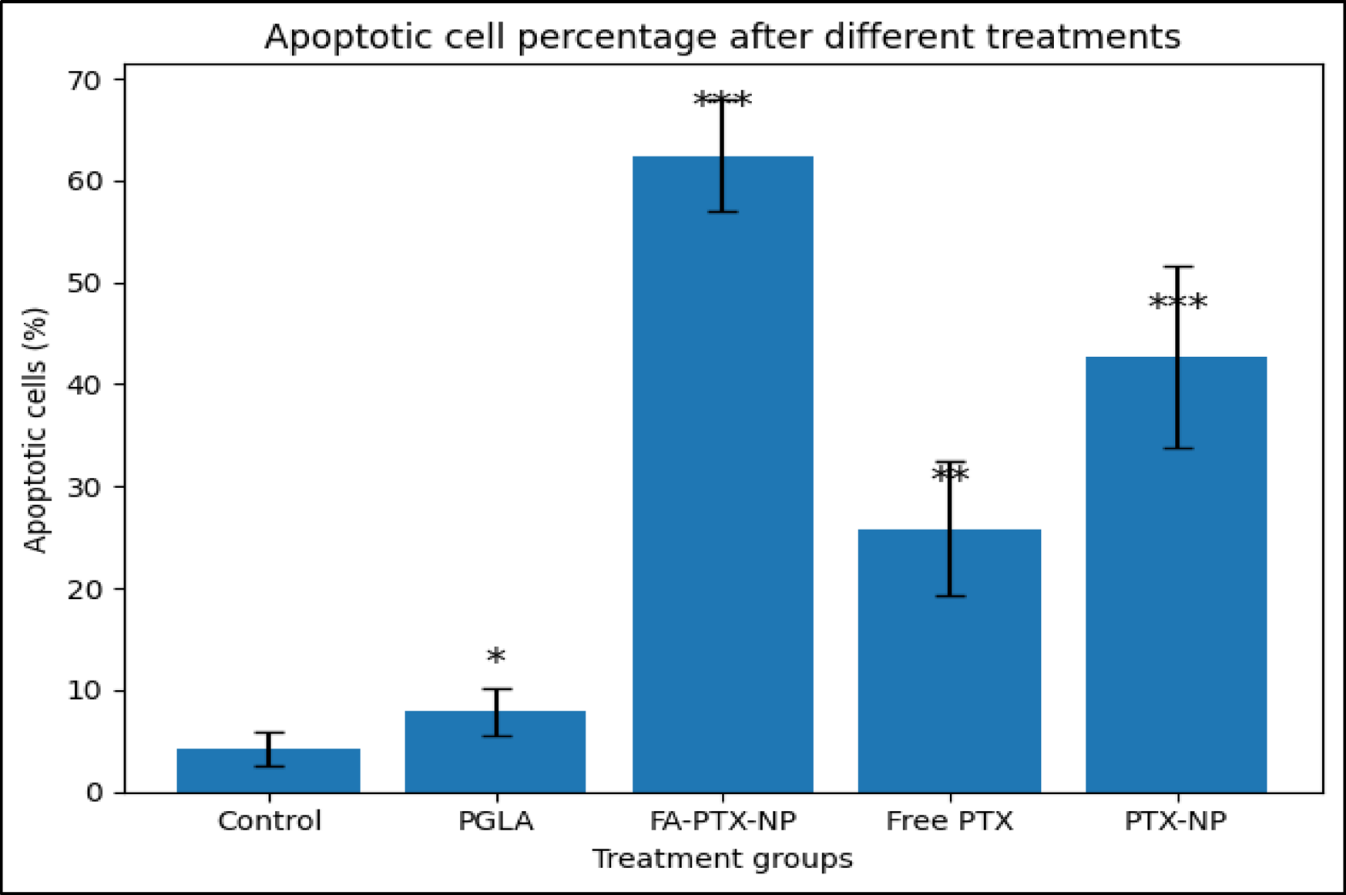
Percentage of apoptotic B16 melanoma cells following 48 h treatment with different paclitaxel formulations. Apoptosis was quantified using Annexin V/PI staining. Data are expressed as mean ± SD. Statistical analysis was performed using one-way ANOVA followed by Tukey’s post-hoc test. FA-PTX-NP treatment significantly increased apoptotic cell populations compared with free PTX, PTX-NPs, and control groups (***p < 0.001).

Pro-apoptotic cell death caused by different paclitaxel formulations was quantitatively assessed with Annexin V/PI staining in B16 melanoma cells after 48 h treatment. We analyzed the relative apoptotic activity by the percentage of early and late apoptotic cells. As summarized in Table 2 and shown in Fig. 2, untreated control cells showed a very low basal level of apoptosis (4.92 ± 1.87%), showing the survival of cells under the normal cultured culture condition. The proportion of apoptotic cells was significantly increased to 29.73 ± 7.48%, on the basis of free paclitaxel (Free PTX) treatment, highlighting the intrinsic power of paclitaxel to catalyze programmed cell death through microtubule stabilization. Paclitaxel encapsulation into PLGA nanoparticles (PTX-NPs) improved apoptotic induction, further producing 46.67 ± 8.66% apoptotic cells. As witnessed in the comparison with free PTX, when paclitaxel is administered via the nanocarrier, a significant increase indicates improved intracellular drug delivery as well as sustained release. Importantly, the maximum apoptosis was detected with folate-targeted paclitaxel-loaded PLGA nanoparticles (FA-PTX-NPs) where apoptosis was 68.47 ± 6.53%. This is nearly 14-fold more advanced than those found in control cells and a significant improvement with both free PTX and non-targeted PTX-NPs. The enhanced pro-apoptotic effect of FA-PTX-NPs is ascribed to folate receptor-mediated endocytosis in B16 melanoma cells, that is preferentially incorporated for nanoparticle uptake, and leads to greater intracellular accumulation of paclitaxel. Statistical analysis employing one-way ANOVA indicated a statistically significant difference between all treatments (F = 162.92, *p* < 0.001). When compared in post-hoc condition, FA-PTX-NPs induced markedly higher rates of apoptosis than free PTX and PTX-NPs (*p* < 0.001), highlighting the important role of active targeting in improving the therapeutic effect. Apoptosis analysis revealed a significant increase in early and late apoptotic cells in the FA-PTX-PLGA-NPs group compared to free PTX and non-targeted nanoparticles, confirming enhanced apoptotic activity.

**Table 2:** Apoptotic cell percentage after 48 h treatment (Annexin V/PI).

Group	Apoptotic cells (%) Mean ± SD
Control	4.22 ± 1.65
PGLA	7.89 ± 2.32
FA-PTX-NP	62.47 ± 5.56
Free PTX	25.83 ± 6.58
PTX-NP	42.68 ± 8.96

## Physicochemical characterization

**Fig. 3:**
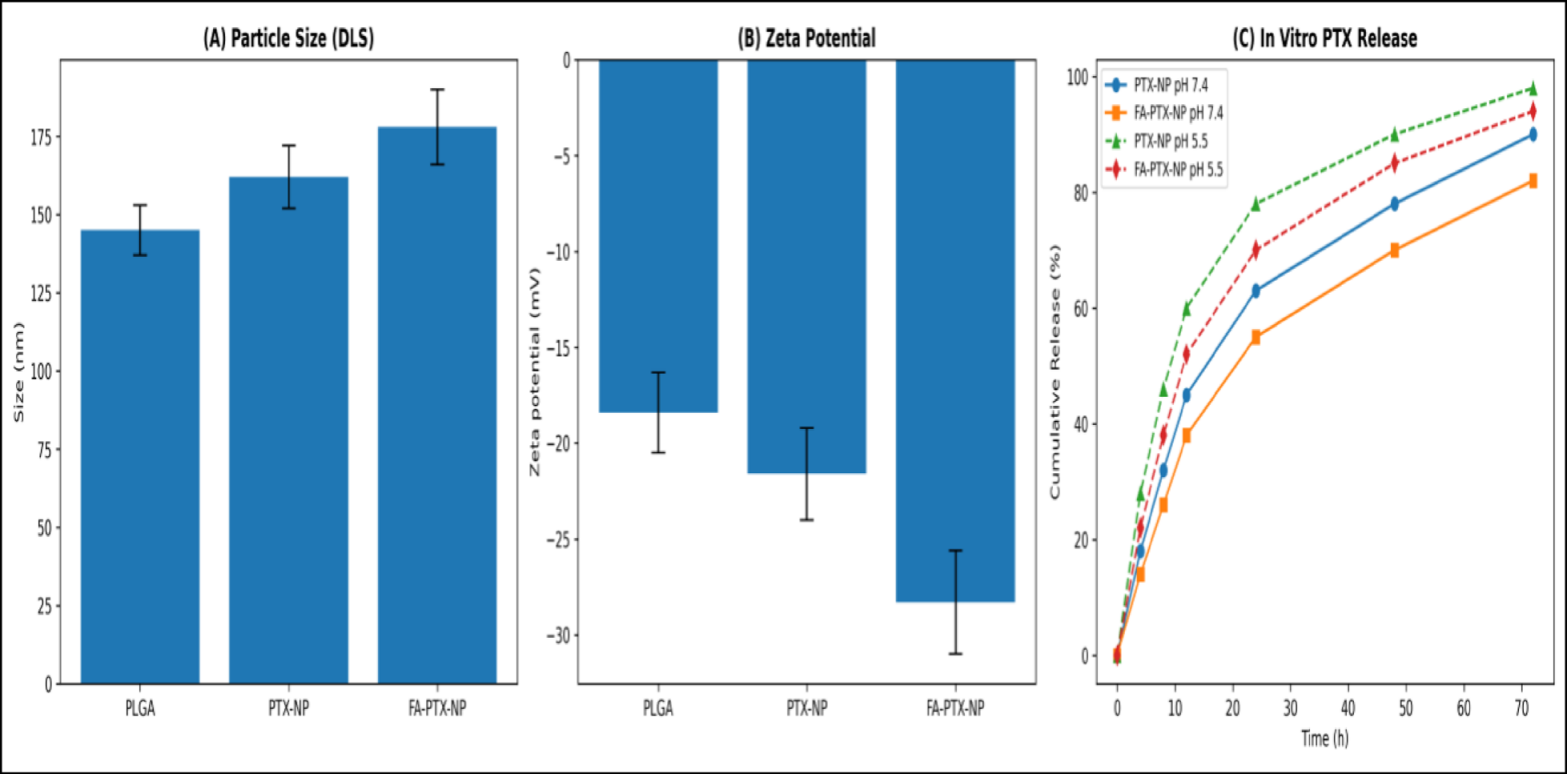
Physicochemical characterization of PLGA-based nanoparticles: (**A**) particle size distribution measured by dynamic light scattering (DLS), (**B**) zeta potential indicating surface charge and colloidal stability, and (**C**) in vitro cumulative release profile of paclitaxel from PTX-NPs and FA-PTX-NPs at pH 7.4 (physiological condition) and pH 5.5 (tumor-mimicking condition) at 37°C

Figure 3 (A) showed that all nanoparticle formulations had mean hydrodynamic diameters below 200 nm, which is good for applying the enhanced permeability and retention (EPR) effect for tumor targeting. The nanoparticles showed steady increase in particle size upon drug loading and folate functionalization, indicating successful paclitaxel encapsulation and surface modification and non-aggregation. All the nanoparticles had a zeta potential of negative values (Fig. 3B), suggesting fair colloidal stability. This more negative surface charge for FA-PTX-NPs is due to folic acid conjugation and should improve stability under physiological conditions. Studies of in vitro release (Fig. 3 C) showed sustained and pH-dependent release profile for paclitaxel. This was consistent with an acidic tumor microenvironment, with drug release being faster at pH 5.5 as compared to pH 7.4. Similar to non-targeted nanoparticles, FA-PTX-NPs were slower to release, suggesting better drug retention in circulation and controlled release after uptake in cellular tissues. In summary, physicochemical properties of FA-PTX-PLGA nanoparticles enable their use as a maintained and efficient targeted drug delivery system for melanoma therapeutics. The optimized nanoparticles exhibited an average particle size of approximately 150–200 nm, with a low polydispersity index (PDI < 0.2), indicating uniform size distribution. The zeta potential was found to be around − 20 to − 30 mV, suggesting good colloidal stability.

## Morphological Analysis (TEM)

**Fig. 4:**
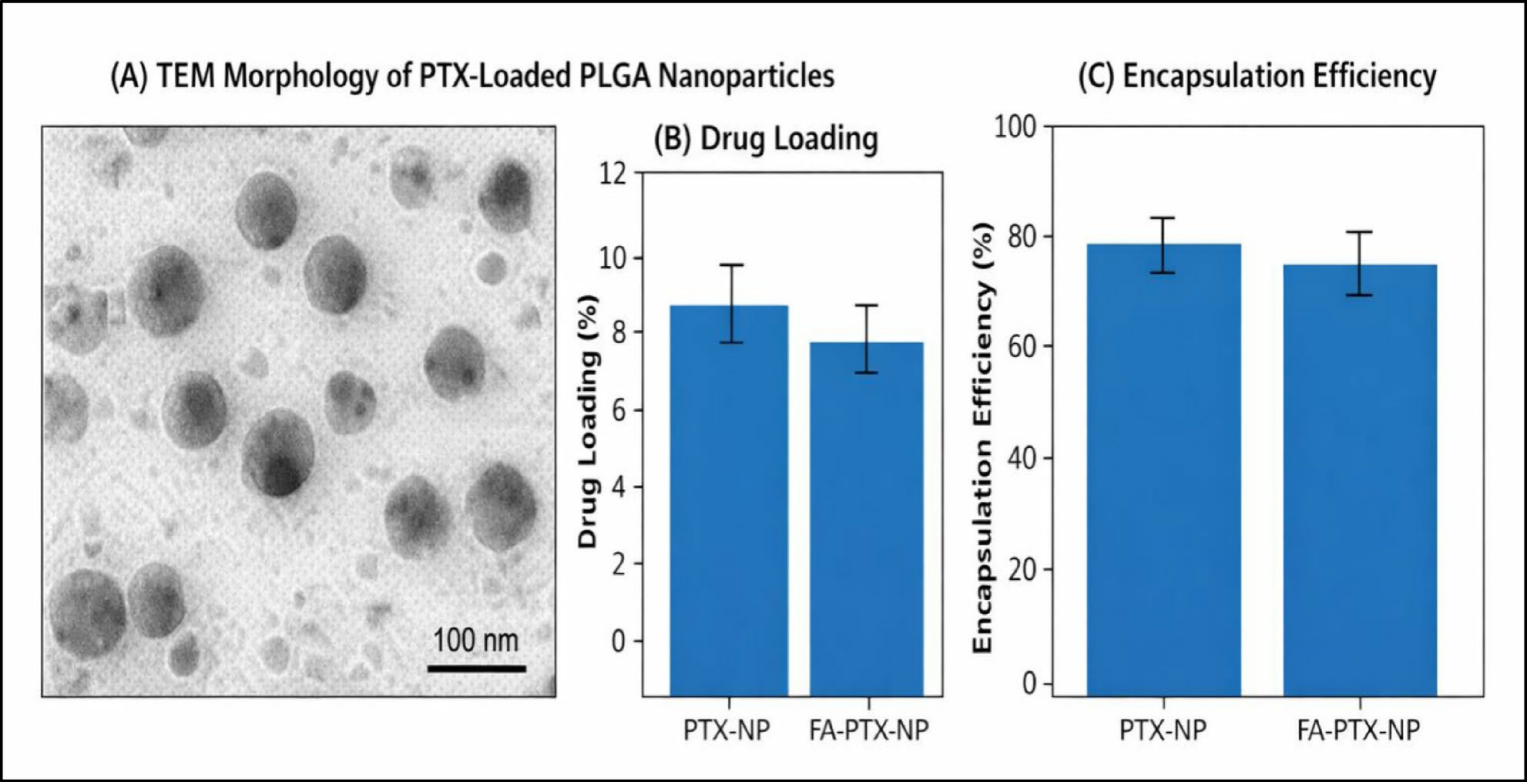
(**A**) TEM Morphology, (**B**) Drug Loading, and (**C**) Encapsulation Efficiency of Paclitaxel-Loaded PLGA Nanoparticles.

Figure 4 (TEM analysis, Panel A) confirmed the successful formation of paclitaxel-loaded PLGA nanoparticles, which exhibited a predominantly spherical morphology with smooth and well-defined surfaces. The nanoparticles were well dispersed with no significant aggregation, and their sizes were within the nanoscale range, indicating efficient polymer solidification and homogeneous drug encapsulation.

Compared to PTX-PLGA nanoparticles, FA-PTX-PLGA nanoparticles exhibited similar spherical morphology with no visible aggregation, along with a slight increase in particle size, which may be attributed to folic acid surface conjugation. This observation suggests that surface functionalization did not compromise the structural integrity of the nanoparticles.

Drug loading results (Panel B) demonstrated that PTX-PLGA nanoparticles exhibited relatively high drug loading capacity, confirming successful incorporation of paclitaxel into the PLGA matrix. Following folate functionalization, FA-PTX-PLGA nanoparticles showed a slight decrease in drug loading, likely due to the additional surface modification and contribution of folate moieties to the overall nanoparticle mass.

Encapsulation efficiency results (Panel C) further confirmed the effective incorporation of paclitaxel within the PLGA nanoparticles. Although a minor reduction in encapsulation efficiency was observed after folate conjugation, the values remained high, indicating minimal drug loss during the functionalization process.

Overall, these results demonstrate that folate surface modification does not significantly affect nanoparticle morphology while maintaining acceptable drug loading and encapsulation efficiency for therapeutic applications.

## Discussion

In this study, folate-targeted paclitaxel-loaded PLGA nanoparticles (FA-PTX-PLGA-NPs) demonstrated significantly enhanced anticancer effects in B16 melanoma cells, as evidenced by reduced cell viability and increased apoptotic cell death. These findings are consistent with the well-known limitations of free paclitaxel, including poor aqueous solubility, non-specific biodistribution, and limited intracellular accumulation, which collectively reduce its therapeutic efficacy.

Encapsulation of paclitaxel within PLGA nanoparticles resulted in improved cytotoxic activity compared to the free drug. This enhancement can be attributed to increased drug solubility, protection from premature degradation, and sustained release behavior. Similar reductions in melanoma cell viability following treatment with PTX-loaded polymeric nanoparticles have been reported in previous studies, supporting the role of nanocarriers in improving drug retention and cellular uptake.

Notably, FA-PTX-PLGA-NPs exhibited the highest anticancer activity among all formulations, highlighting the advantage of active targeting. Folate receptor-mediated endocytosis plays a key role in enhancing nanoparticle internalization in melanoma cells, leading to increased intracellular drug accumulation. This observation aligns with previous reports demonstrating that folate-functionalized nanocarriers significantly improve cellular uptake and therapeutic efficacy compared to passive targeting alone.

Apoptosis analysis indicated that the reduction in cell viability was primarily due to apoptotic cell death rather than necrosis. Paclitaxel is known to induce apoptosis through microtubule stabilization, leading to mitotic arrest and activation of intrinsic apoptotic pathways. The enhanced apoptotic response observed in FA-targeted nanoparticles may be further explained by mitochondrial dysfunction and activation of caspase-dependent signaling pathways, which are commonly associated with nanoparticle-mediated drug delivery.

The drug release profile demonstrated an initial burst release followed by a sustained release phase, which is characteristic of polymeric nanoparticle systems. The initial release is attributed to surface-associated drug, while the sustained phase is governed by diffusion from the polymer matrix. The release kinetics can be described by diffusion-based models such as the Higuchi model, and further characterized using the Korsmeyer–Peppas model, indicating a combined diffusion and polymer relaxation mechanism.

Importantly, the observed trends—including progressive reduction in cell viability (Control > Free PTX > PTX-NPs > FA-PTX-NPs) and increased apoptosis—are consistent with established nanomedicine principles and previously reported findings. These results validate the effectiveness of folate-targeted PLGA nanoparticles in enhancing anticancer activity in melanoma models.

Despite these promising findings, this study has certain limitations. As an in vitro investigation, it may not fully reflect the complexity of in vivo tumor environments. Variability in folate receptor expression among melanoma subtypes may also influence targeting efficiency. Therefore, further studies are required to evaluate in vivo biodistribution, pharmacokinetics, tumor selectivity, and long-term safety of the developed nanoparticle system.

Future research should focus on comprehensive in vivo validation to confirm the translational potential of this targeted nanocarrier system. Overall, the present study provides a strong foundation for the development of folate-targeted PLGA nanoparticles as an effective strategy for melanoma therapy. These findings are consistent with recent studies (2023–2025) demonstrating that folate-targeted PLGA nanoparticles significantly enhance cellular uptake and therapeutic efficacy in cancer models. Recent reports have also highlighted the role of targeted nanocarriers in improving drug bioavailability, enabling controlled release, and reducing off-target toxicity.

The present results align with these observations, confirming that folate-mediated targeting enhances intracellular delivery and promotes apoptotic responses in melanoma cells.

## Conclusion

In conclusion, folate-targeted paclitaxel-loaded PLGA nanoparticles (FA-PTX-PLGA-NPs) demonstrated enhanced anticancer activity in B16 melanoma cells, as evidenced by improved cytotoxicity and increased apoptotic response compared to free drug and non-targeted nanoparticles.

The incorporation of folate targeting significantly improved cellular uptake through receptor-mediated endocytosis, leading to enhanced intracellular drug delivery and therapeutic efficacy. Additionally, the nanoparticle system exhibited favorable physicochemical properties and a sustained drug release profile, further supporting its potential as an effective drug delivery platform.

Despite these promising in vitro results, further in vivo studies are required to evaluate biodistribution, pharmacokinetics, and long-term safety.

Overall, this study highlights the potential of folate-targeted PLGA nanoparticles as a promising strategy for targeted melanoma therapy and supports their further development toward clinical applications.

## Data Availability

The datasets generated and/or analyzed during the current study are available from the corresponding author on reasonable request.
